# Supplementary material for: Leucobacter manosquensis sp. nov.—A Novel Bacterial Species Isolated from Healthy Human Skin
Source: Microorganisms. 2023 Oct 11;11(10):2535. doi: 10.3390/microorganisms11102535 (PMC10609233; doi:10.3390/microorganisms11102535)
Supplement: Supplementary file 1 [file microorganisms-11-02535-s001.zip › microorganisms-2604571-supplementary.pptx]

## Slide 1
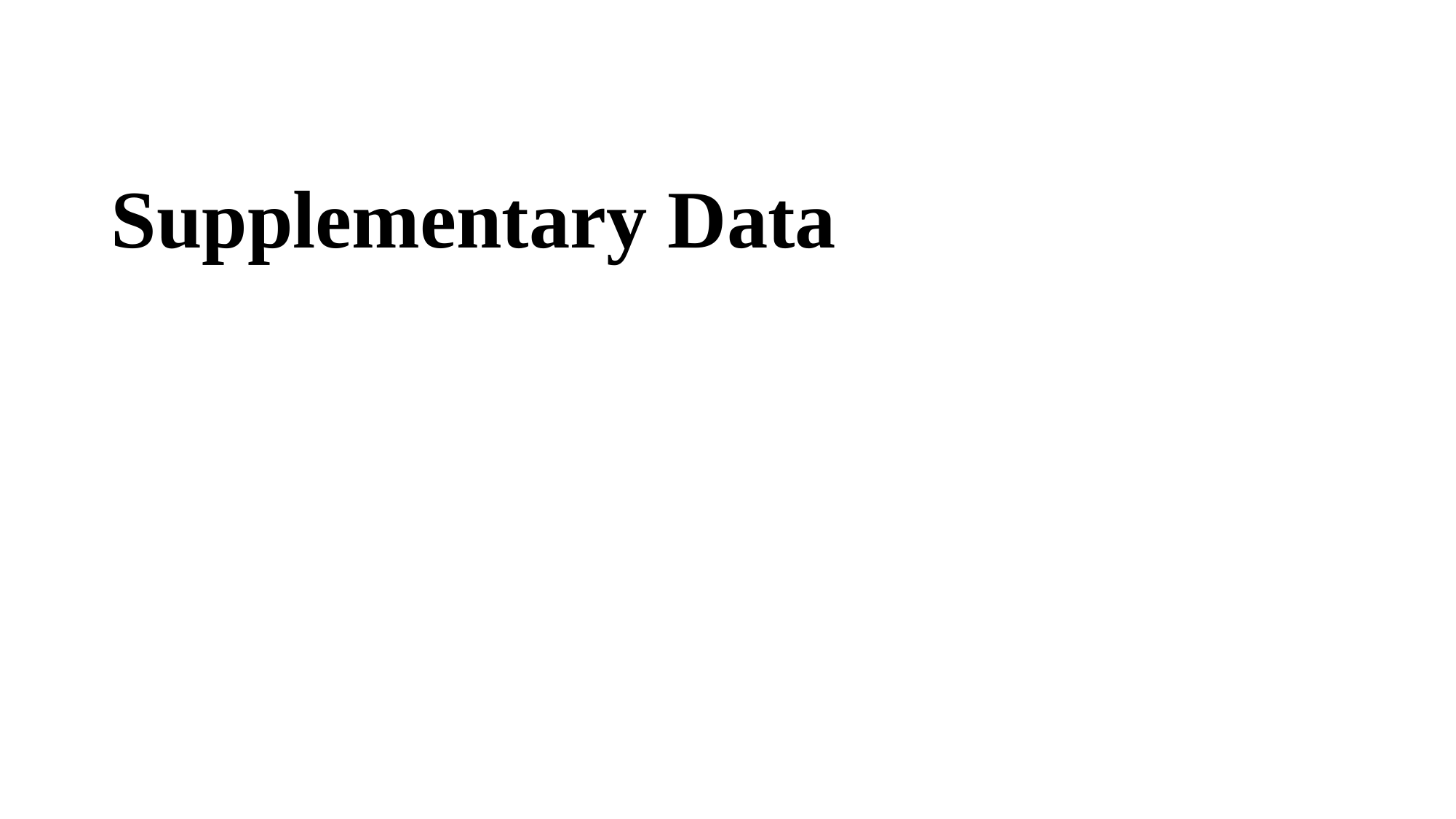

Supplementary Data

## Slide 2
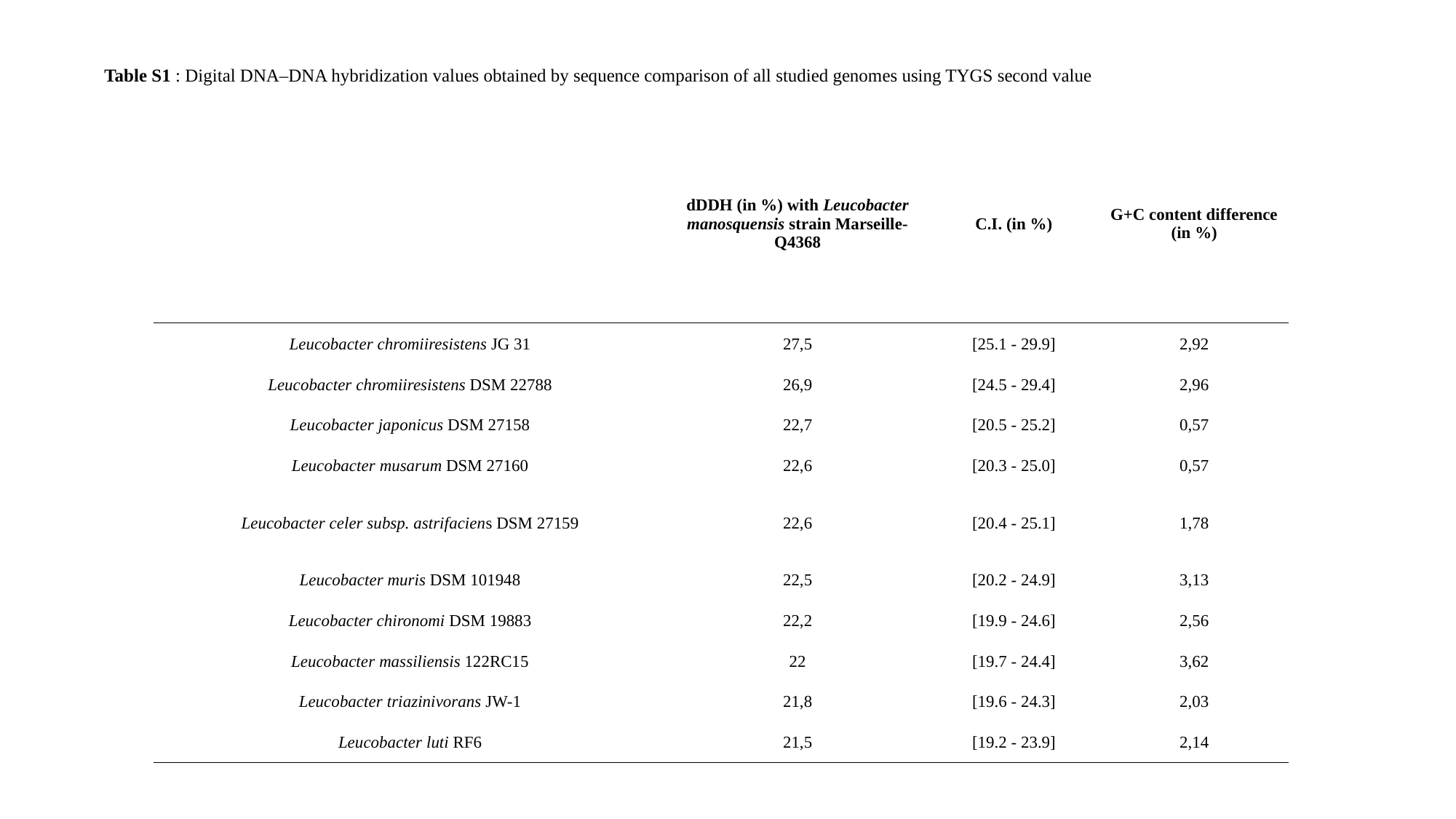

Table S1 : Digital DNA–DNA hybridization values obtained by sequence comparison of all studied genomes using TYGS second value
| | dDDH (in %) with Leucobacter manosquensis strain Marseille-Q4368 | C.I. (in %) | G+C content difference (in %) |
| --- | --- | --- | --- |
| Leucobacter chromiiresistens JG 31 | 27,5 | [25.1 - 29.9] | 2,92 |
| Leucobacter chromiiresistens DSM 22788 | 26,9 | [24.5 - 29.4] | 2,96 |
| Leucobacter japonicus DSM 27158 | 22,7 | [20.5 - 25.2] | 0,57 |
| Leucobacter musarum DSM 27160 | 22,6 | [20.3 - 25.0] | 0,57 |
| Leucobacter celer subsp. astrifaciens DSM 27159 | 22,6 | [20.4 - 25.1] | 1,78 |
| Leucobacter muris DSM 101948 | 22,5 | [20.2 - 24.9] | 3,13 |
| Leucobacter chironomi DSM 19883 | 22,2 | [19.9 - 24.6] | 2,56 |
| Leucobacter massiliensis 122RC15 | 22 | [19.7 - 24.4] | 3,62 |
| Leucobacter triazinivorans JW-1 | 21,8 | [19.6 - 24.3] | 2,03 |
| Leucobacter luti RF6 | 21,5 | [19.2 - 23.9] | 2,14 |

## Slide 3
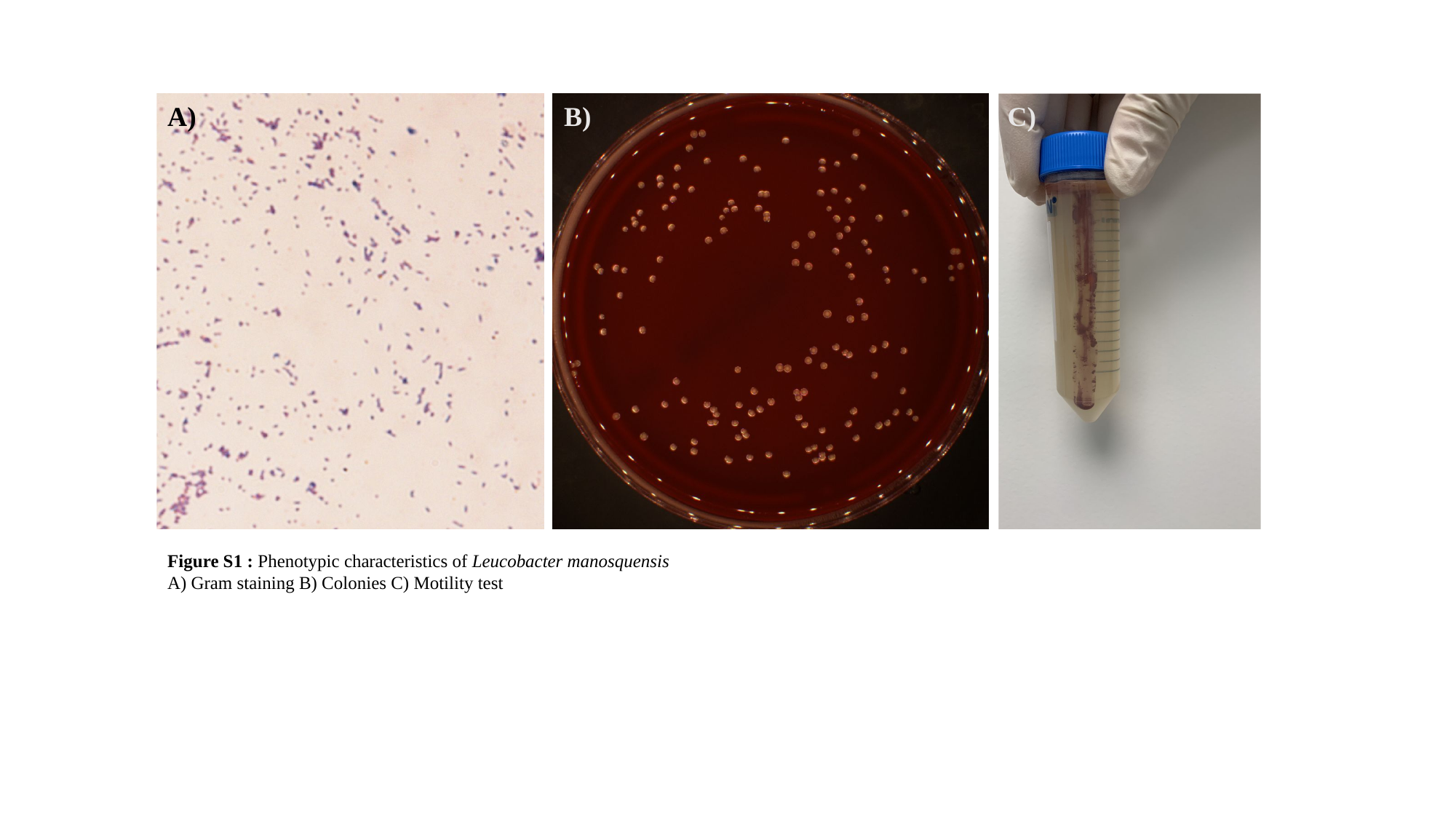

A)			          B)				           C)
Figure S1 : Phenotypic characteristics of Leucobacter manosquensis
A) Gram staining B) Colonies C) Motility test

## Slide 4
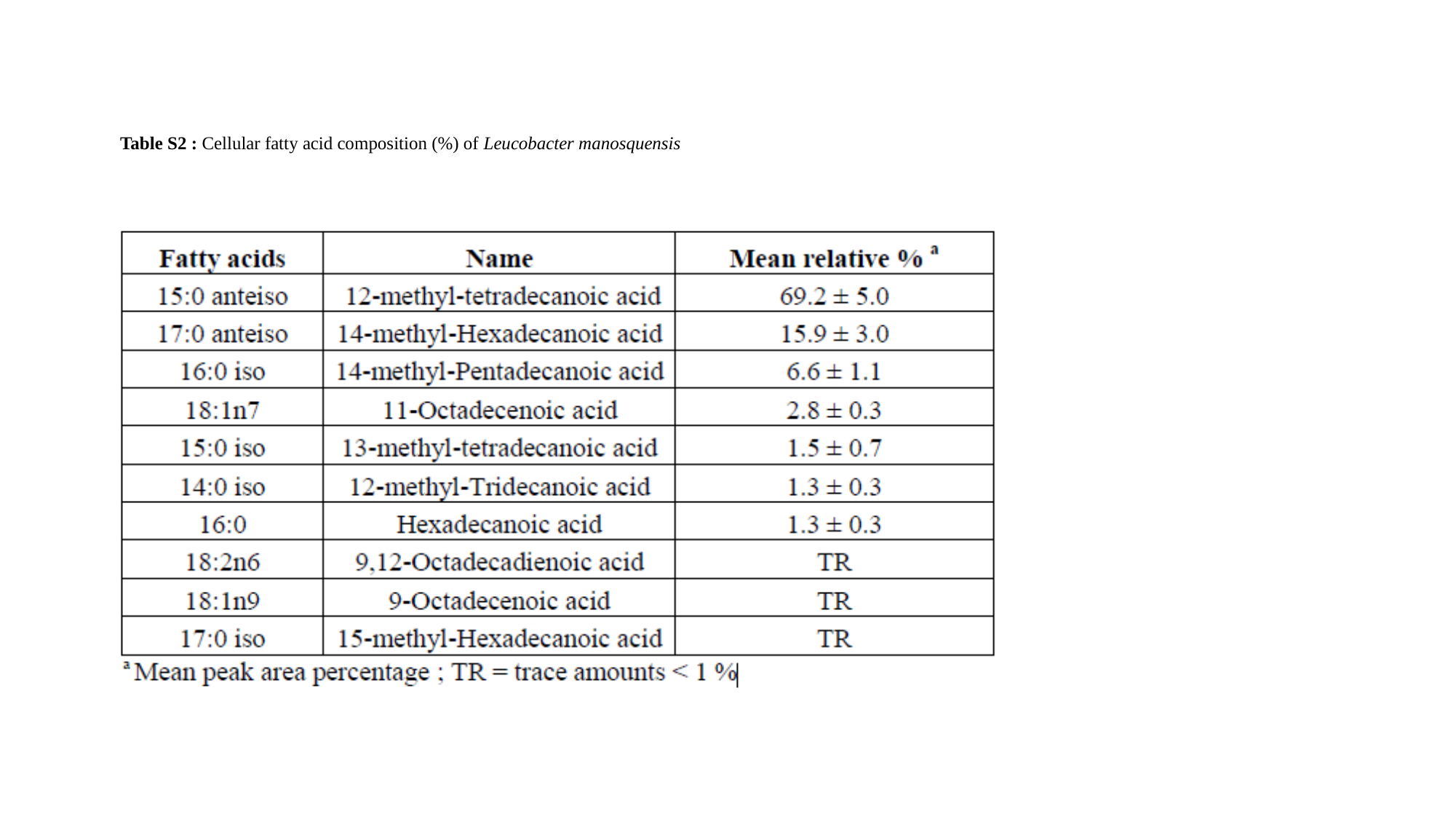

Table S2 : Cellular fatty acid composition (%) of Leucobacter manosquensis

## Slide 5
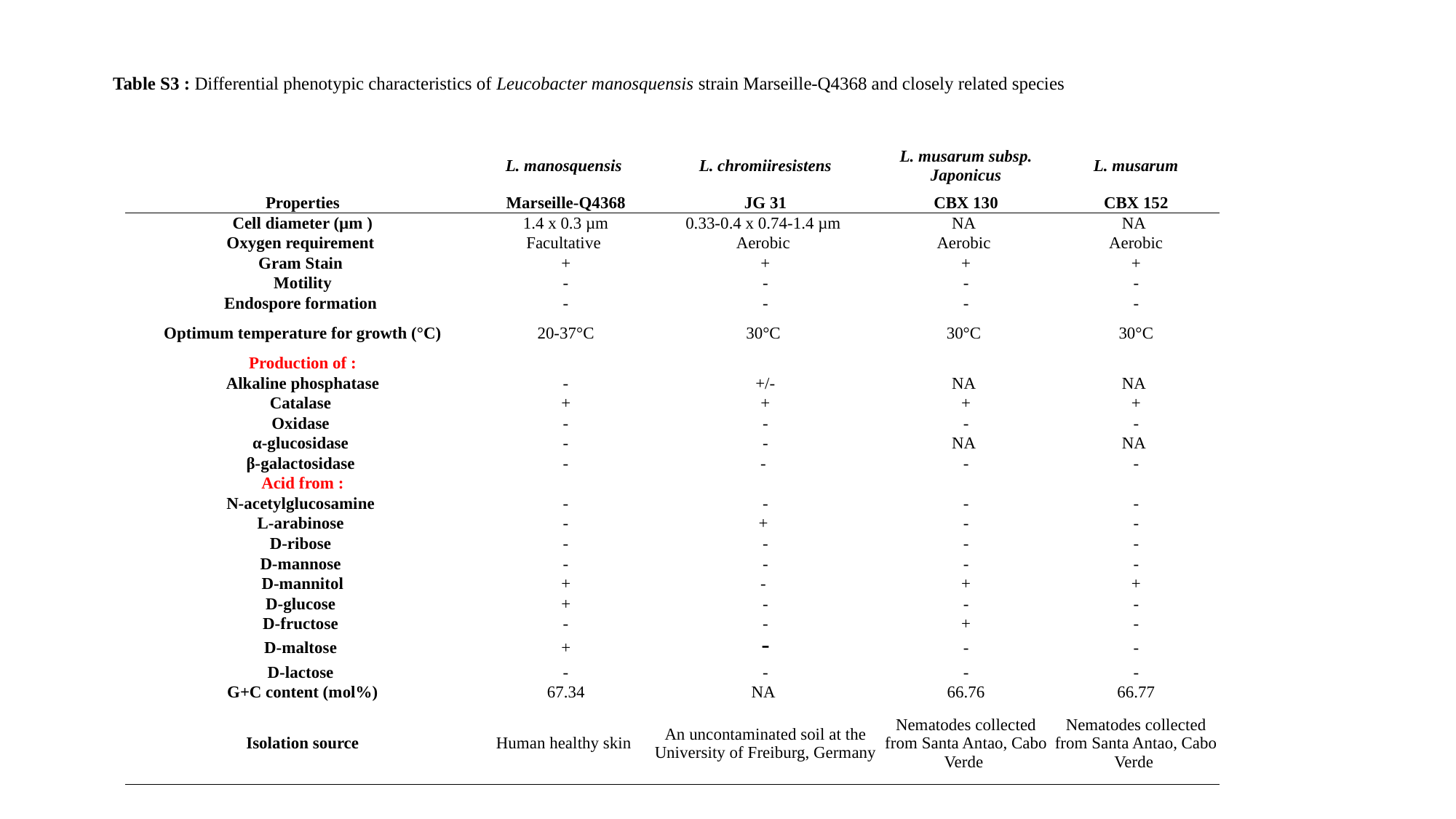

Table S3 : Differential phenotypic characteristics of Leucobacter manosquensis strain Marseille-Q4368 and closely related species
| | L. manosquensis | L. chromiiresistens | L. musarum subsp. Japonicus | L. musarum |
| --- | --- | --- | --- | --- |
| Properties | Marseille-Q4368 | JG 31 | CBX 130 | CBX 152 |
| Cell diameter (µm ) | 1.4 x 0.3 µm | 0.33-0.4 x 0.74-1.4 µm | NA | NA |
| Oxygen requirement | Facultative | Aerobic | Aerobic | Aerobic |
| Gram Stain | + | + | + | + |
| Motility | - | - | - | - |
| Endospore formation | - | - | - | - |
| Optimum temperature for growth (°C) | 20-37°C | 30°C | 30°C | 30°C |
| Production of : | | | | |
| Alkaline phosphatase | - | +/- | NA | NA |
| Catalase | + | + | + | + |
| Oxidase | - | - | - | - |
| α-glucosidase | - | - | NA | NA |
| β-galactosidase | - | - | - | - |
| Acid from : | | | | |
| N-acetylglucosamine | - | - | - | - |
| L-arabinose | - | + | - | - |
| D-ribose | - | - | - | - |
| D-mannose | - | - | - | - |
| D-mannitol | + | - | + | + |
| D-glucose | + | - | - | - |
| D-fructose | - | - | + | - |
| D-maltose | + | - | - | - |
| D-lactose | - | - | - | - |
| G+C content (mol%) | 67.34 | NA | 66.76 | 66.77 |
| Isolation source | Human healthy skin | An uncontaminated soil at the University of Freiburg, Germany | Nematodes collected from Santa Antao, Cabo Verde | Nematodes collected from Santa Antao, Cabo Verde |

## Slide 6
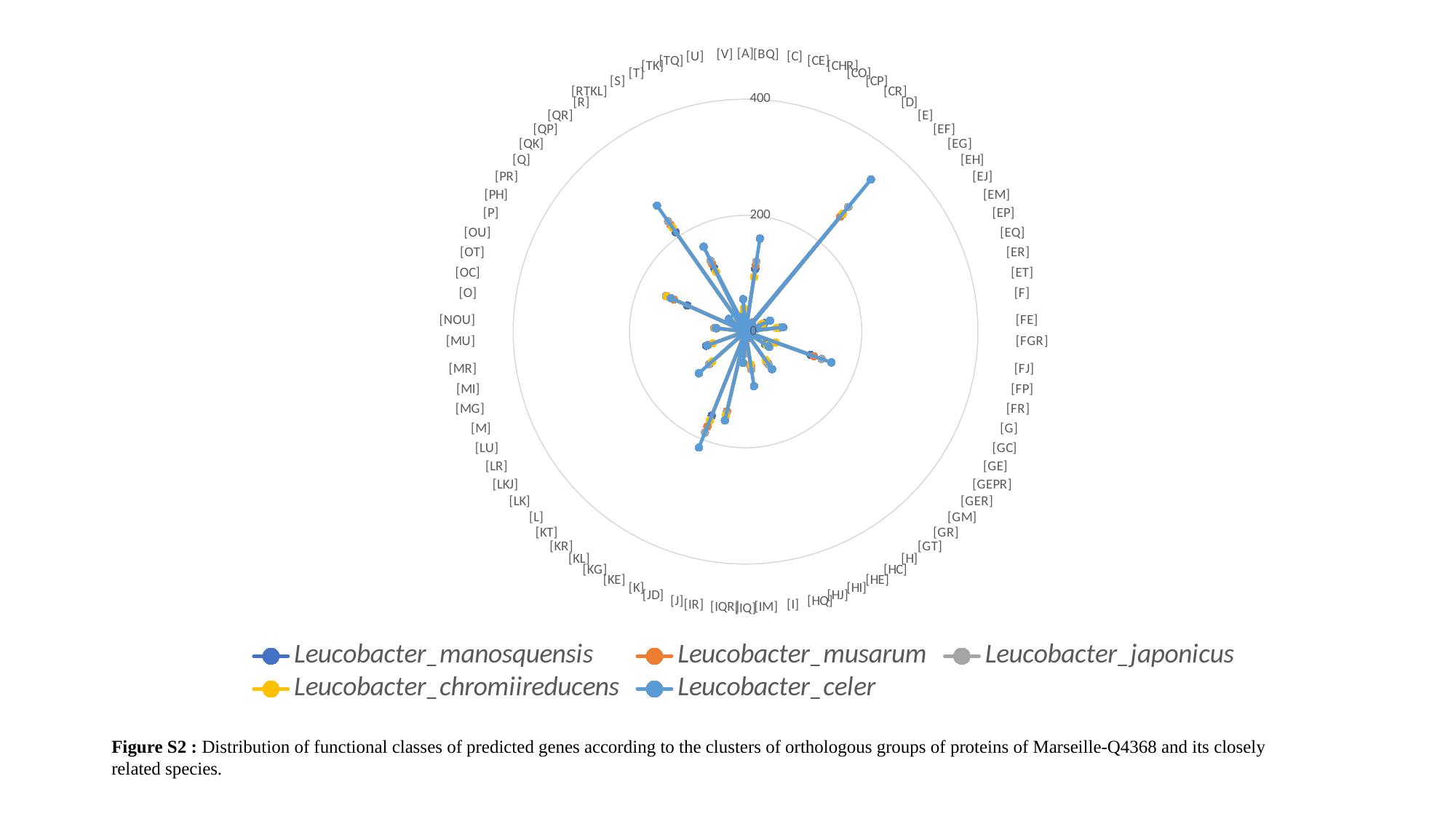

### Chart
| Category | Leucobacter_manosquensis | Leucobacter_musarum | Leucobacter_japonicus | Leucobacter_chromiireducens | Leucobacter_celer |
|---|---|---|---|---|---|
| [A] | 1.0 | 1.0 | 1.0 | 1.0 | 1.0 |
| [BQ] | 1.0 | 0.0 | 0.0 | 0.0 | 1.0 |
| [C] | 109.0 | 115.0 | 122.0 | 95.0 | 162.0 |
| [CE] | 2.0 | 2.0 | 2.0 | 2.0 | 3.0 |
| [CHR] | 0.0 | 0.0 | 1.0 | 0.0 | 0.0 |
| [CO] | 2.0 | 2.0 | 2.0 | 2.0 | 2.0 |
| [CP] | 1.0 | 2.0 | 2.0 | 2.0 | 3.0 |
| [CR] | 2.0 | 5.0 | 5.0 | 1.0 | 4.0 |
| [D] | 18.0 | 19.0 | 16.0 | 15.0 | 18.0 |
| [E] | 262.0 | 256.0 | 278.0 | 263.0 | 339.0 |
| [EF] | 2.0 | 2.0 | 2.0 | 2.0 | 2.0 |
| [EG] | 1.0 | 3.0 | 3.0 | 2.0 | 1.0 |
| [EH] | 12.0 | 11.0 | 12.0 | 11.0 | 12.0 |
| [EJ] | 1.0 | 2.0 | 2.0 | 1.0 | 2.0 |
| [EM] | 4.0 | 3.0 | 3.0 | 2.0 | 3.0 |
| [EP] | 35.0 | 30.0 | 32.0 | 32.0 | 46.0 |
| [EQ] | 6.0 | 4.0 | 9.0 | 5.0 | 9.0 |
| [ER] | 12.0 | 10.0 | 13.0 | 7.0 | 21.0 |
| [ET] | 9.0 | 6.0 | 10.0 | 12.0 | 12.0 |
| [F] | 65.0 | 58.0 | 57.0 | 54.0 | 64.0 |
| [FE] | 1.0 | 1.0 | 1.0 | 1.0 | 1.0 |
| [FGR] | 2.0 | 2.0 | 2.0 | 2.0 | 2.0 |
| [FJ] | 2.0 | 2.0 | 2.0 | 3.0 | 1.0 |
| [FP] | 1.0 | 1.0 | 1.0 | 1.0 | 1.0 |
| [FR] | 4.0 | 5.0 | 4.0 | 4.0 | 4.0 |
| [G] | 119.0 | 125.0 | 139.0 | 55.0 | 157.0 |
| [GC] | 1.0 | 1.0 | 0.0 | 1.0 | 0.0 |
| [GE] | 0.0 | 0.0 | 0.0 | 0.0 | 1.0 |
| [GEPR] | 40.0 | 46.0 | 49.0 | 44.0 | 47.0 |
| [GER] | 1.0 | 2.0 | 3.0 | 4.0 | 3.0 |
| [GM] | 3.0 | 4.0 | 4.0 | 3.0 | 3.0 |
| [GR] | 0.0 | 0.0 | 0.0 | 0.0 | 1.0 |
| [GT] | 0.0 | 1.0 | 0.0 | 0.0 | 0.0 |
| [H] | 67.0 | 62.0 | 69.0 | 60.0 | 79.0 |
| [HC] | 3.0 | 2.0 | 2.0 | 2.0 | 3.0 |
| [HE] | 5.0 | 12.0 | 11.0 | 7.0 | 11.0 |
| [HI] | 1.0 | 1.0 | 1.0 | 1.0 | 1.0 |
| [HJ] | 1.0 | 1.0 | 1.0 | 1.0 | 1.0 |
| [HQ] | 0.0 | 1.0 | 0.0 | 0.0 | 1.0 |
| [I] | 60.0 | 62.0 | 66.0 | 57.0 | 95.0 |
| [IM] | 1.0 | 1.0 | 1.0 | 1.0 | 1.0 |
| [IQ] | 7.0 | 4.0 | 8.0 | 6.0 | 6.0 |
| [IQR] | 20.0 | 30.0 | 39.0 | 18.0 | 54.0 |
| [IR] | 3.0 | 2.0 | 2.0 | 2.0 | 4.0 |
| [J] | 143.0 | 141.0 | 144.0 | 148.0 | 157.0 |
| [JD] | 0.0 | 1.0 | 0.0 | 0.0 | 0.0 |
| [K] | 156.0 | 176.0 | 187.0 | 165.0 | 215.0 |
| [KE] | 3.0 | 2.0 | 2.0 | 6.0 | 3.0 |
| [KG] | 4.0 | 5.0 | 5.0 | 2.0 | 8.0 |
| [KL] | 4.0 | 4.0 | 2.0 | 2.0 | 2.0 |
| [KR] | 6.0 | 10.0 | 12.0 | 7.0 | 8.0 |
| [KT] | 6.0 | 6.0 | 5.0 | 3.0 | 2.0 |
| [L] | 83.0 | 84.0 | 84.0 | 77.0 | 108.0 |
| [LK] | 2.0 | 2.0 | 2.0 | 2.0 | 2.0 |
| [LKJ] | 4.0 | 4.0 | 4.0 | 4.0 | 4.0 |
| [LR] | 3.0 | 3.0 | 3.0 | 3.0 | 3.0 |
| [LU] | 2.0 | 1.0 | 3.0 | 3.0 | 2.0 |
| [M] | 72.0 | 68.0 | 69.0 | 60.0 | 70.0 |
| [MG] | 6.0 | 8.0 | 6.0 | 3.0 | 5.0 |
| [MI] | 1.0 | 1.0 | 1.0 | 1.0 | 2.0 |
| [MR] | 2.0 | 2.0 | 2.0 | 3.0 | 1.0 |
| [MU] | 1.0 | 1.0 | 1.0 | 2.0 | 1.0 |
| [NOU] | 0.0 | 0.0 | 0.0 | 0.0 | 1.0 |
| [O] | 52.0 | 54.0 | 53.0 | 50.0 | 51.0 |
| [OC] | 3.0 | 2.0 | 2.0 | 6.0 | 3.0 |
| [OT] | 1.0 | 1.0 | 1.0 | 1.0 | 1.0 |
| [OU] | 2.0 | 2.0 | 2.0 | 2.0 | 2.0 |
| [P] | 110.0 | 135.0 | 150.0 | 148.0 | 141.0 |
| [PH] | 1.0 | 8.0 | 11.0 | 5.0 | 6.0 |
| [PR] | 1.0 | 1.0 | 1.0 | 1.0 | 1.0 |
| [Q] | 21.0 | 17.0 | 22.0 | 17.0 | 36.0 |
| [QK] | 0.0 | 1.0 | 1.0 | 0.0 | 0.0 |
| [QP] | 0.0 | 0.0 | 0.0 | 1.0 | 0.0 |
| [QR] | 3.0 | 3.0 | 3.0 | 2.0 | 4.0 |
| [R] | 209.0 | 225.0 | 232.0 | 219.0 | 265.0 |
| [RTKL] | 2.0 | 2.0 | 4.0 | 2.0 | 2.0 |
| [S] | 122.0 | 130.0 | 136.0 | 115.0 | 163.0 |
| [T] | 26.0 | 23.0 | 21.0 | 30.0 | 27.0 |
| [TK] | 19.0 | 19.0 | 17.0 | 22.0 | 26.0 |
| [TQ] | 3.0 | 5.0 | 3.0 | 6.0 | 7.0 |
| [U] | 15.0 | 11.0 | 12.0 | 14.0 | 16.0 |
| [V] | 31.0 | 38.0 | 37.0 | 40.0 | 56.0 |Figure S2 : Distribution of functional classes of predicted genes according to the clusters of orthologous groups of proteins of Marseille-Q4368 and its closely related species.

## Slide 7
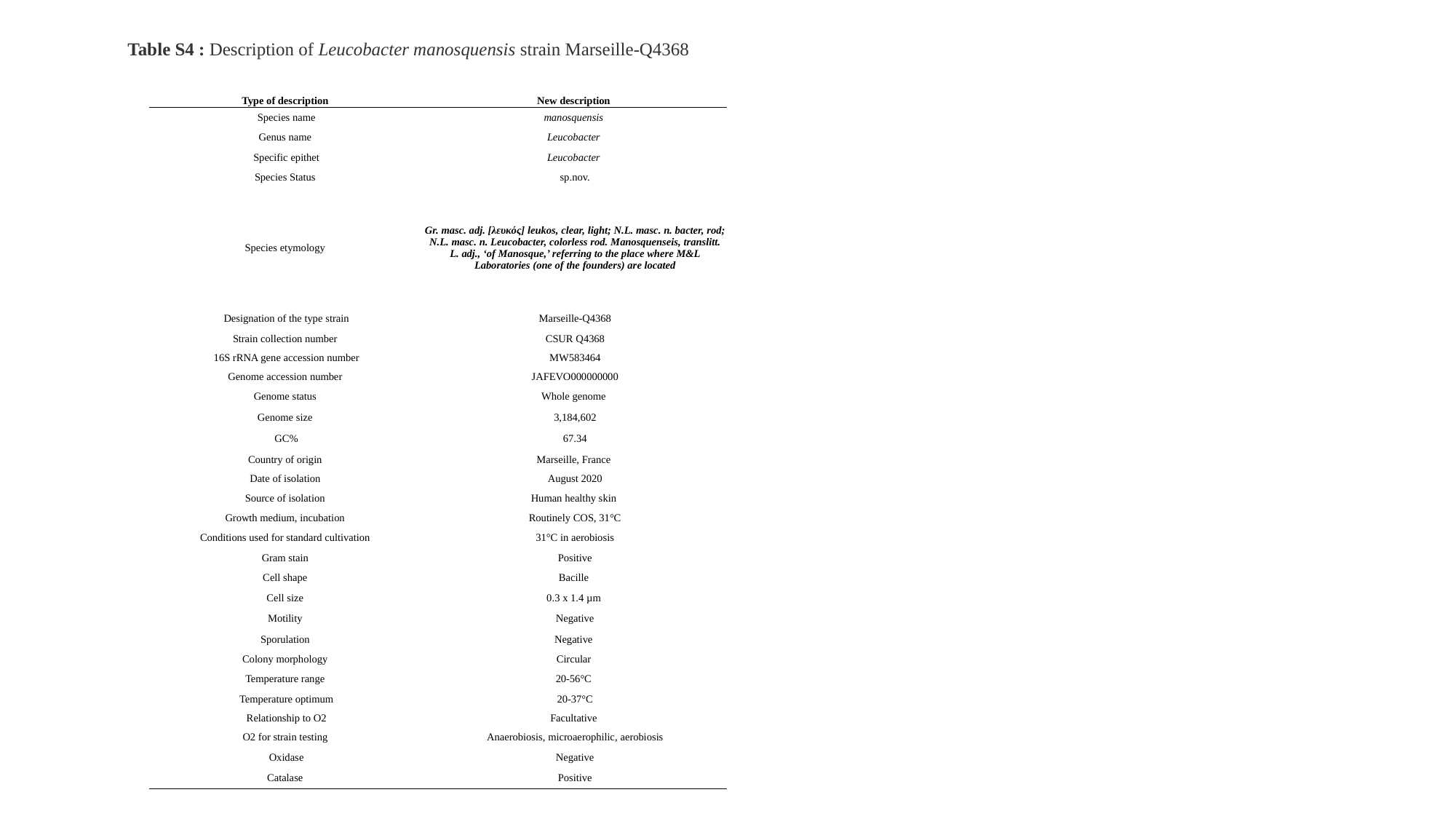

Table S4 : Description of Leucobacter manosquensis strain Marseille-Q4368
| Type of description | New description |
| --- | --- |
| Species name | manosquensis |
| Genus name | Leucobacter |
| Specific epithet | Leucobacter |
| Species Status | sp.nov. |
| Species etymology | Gr. masc. adj. [λευκός] leukos, clear, light; N.L. masc. n. bacter, rod; N.L. masc. n. Leucobacter, colorless rod. Manosquenseis, translitt. L. adj., ‘of Manosque,’ referring to the place where M&L Laboratories (one of the founders) are located |
| Designation of the type strain | Marseille-Q4368 |
| Strain collection number | CSUR Q4368 |
| 16S rRNA gene accession number | MW583464 |
| Genome accession number | JAFEVO000000000 |
| Genome status | Whole genome |
| Genome size | 3,184,602 |
| GC% | 67.34 |
| Country of origin | Marseille, France |
| Date of isolation | August 2020 |
| Source of isolation | Human healthy skin |
| Growth medium, incubation | Routinely COS, 31°C |
| Conditions used for standard cultivation | 31°C in aerobiosis |
| Gram stain | Positive |
| Cell shape | Bacille |
| Cell size | 0.3 x 1.4 µm |
| Motility | Negative |
| Sporulation | Negative |
| Colony morphology | Circular |
| Temperature range | 20-56°C |
| Temperature optimum | 20-37°C |
| Relationship to O2 | Facultative |
| O2 for strain testing | Anaerobiosis, microaerophilic, aerobiosis |
| Oxidase | Negative |
| Catalase | Positive |
